# Erythema caused by a localised skin infection with *Arthrobacter mysorens*

**DOI:** 10.1186/1471-2334-10-352

**Published:** 2010-12-15

**Authors:** Can Imirzalioglu, Torsten Hain, Hamid Hossain, Trinad Chakraborty, Eugen Domann

**Affiliations:** 1 Institute of Medical Microbiology, Justus-Liebig-University Giessen, Frankfurter Strasse 107, D-35392 Giessen, Germany

## Abstract

**Background:**

Skin erythemas of unknown origin are a frequent reason for consulting the general practitioner or dermatologist.

**Case presentation:**

Here we report a case of an erythema resembling the erythema migrans manifestation of Lyme disease, but with atypical symptoms like persistent pruritus. The patient had no history of a recent tick-bite but displayed a positive serology for an advanced stage of Lyme borreliosis, which stood in contrast to the clinical manifestation of erythema migrans as a symptom of early Lyme disease. Three skin swabs and soil samples, collected in the area where the patient possibly acquired the infection, were examined by bacterial and fungal culture methods. Microorganisms were identified by using 16 S rRNA gene sequencing and bioinformatics. The patient and soil isolates were compared by employing RAPD analysis. The serum samples of the patient were examined by immunoblotting. *Arthrobacter mysorens*, a soil bacterium, was isolated from the collected skin and soil samples. The identity of both isolates was determined by molecular fingerprinting methods. *A. mysorens *was proven to be causative for the erythema by direct isolation from the affected skin and a positive serology, thus explaining the atypical appearance of the erythema compared to erythema migrans caused by *Borrelia *infection.

**Conclusions:**

Infections with A.my*sorens *might be underreported and microbiological diagnostic techniques should be applied in cases of patients with unclear erythemas, resembling erythema migrans, without a history of tick bites.

## Background

Skin erythemas of unknown origin are a frequent reason for consulting the general practitioner or dermatologist. Among many clinicians, laminary spreading erythemas often lead to the diagnosis of a tick bite-associated erythema migrans (EM), a symptom of early localized infection with *Borrelia burgdorferi (sensu lato) *[1,2]. As the development of an immunologic response to this infection usually takes 4 to 6 weeks and the incubation period for EM is typically 7 to 14 days, early Lyme borreliosis often presents itself with a negative serology [3,4]. In addition, tick bites are not always described or remembered by the patient. Thus, the diagnosis is mostly based on clinical symptoms. In its typical appearance, EM is a homogenous spreading, indolent, erythematous, oval shaped lesion with a bright red border and a central clearing. Minimal pruritus might be present at an early stage. EM develops at the site of the tick bite and therefore can be located on any part of the body. Mild systemic symptoms like low-grade fever and chills might be present. EM in the United States is often associated with more prominent signs of inflammation, as compared to that in Europe [1-4]. This case report illustrates that erythemas caused by other pathogens might resemble this clinical picture, thus a false diagnosis may be made which may complicate and prolong the disease process and prevent adequate therapy.

### Case presentation

**Figure 1:**
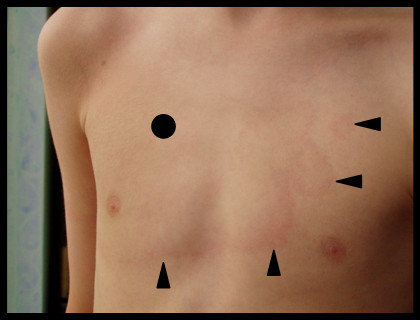
**Erythema on the patient's chest**. The black dot indicates the initial focus of the erythema, the arrow heads the periphery of the lesion. *Arthrobacter mysorens *was isolated from the black dot area and from the red edge close to the arrow heads.

In June early summer, a nine-year old boy spent four hours in a forest digging out a bicycle track to ride his mountain bike. He returned home with a dirty shirt in particular at the right side of the chest, very close to the right acromastium. Since he felt a localised pruritus there, he had intensively scratched the region, thereby contaminating the skin with forest soil. A small erythema with an average diameter of one centimetre and a clear-cut red edge above the right acromastium was apparent on the following day. His mother suspected a potential insect or tick bite, although no tick could be found. The patient had no previous erosion. The then conducted *Borrelia*-specific ELISA was negative for IgM antibodies but positive for IgG antibodies. An immunoblot (Recomblot Borrelia, Mikrogen, Germany) with the patient's serum revealed a *B. burgdorferi sensu lato*-specific, IgG antibody response to p100, p41, BmpA, OspC (weakly positive), p41, and p18 but no IgM-specific antibody response could be detected. This finding was consistent with a *Borrelia *infection at an advanced stage (> 6 months after infection) or a residual of an earlier infection, as a symptom-free patient may also have a similar *Borrelia*-specific antibody response based on longtime persistent antibodies. Clinical findings in this stage are typically those of advanced neuroborreliosis (progressive encephalomyelitis etc.), acrodermatitis chronica atrophicans or Lyme arthritis. Since the patient did not display any symptoms corresponding to these clinical syndromes, a residual, asymptomatic infection was suspected and no specific treatment was initiated. These findings argue against a *Borrelia *infection as the cause for the patient's symptoms, as EM caused by *Borrelia burgdorferi s. l*. represents a very early stage of infection [2,5,6] and would probably lead to a specific IgM antibody response. To exclude a possible re-infection, a second serum sample parallel to the first sample was tested 4 weeks later. However, no significant serological changes could be observed. Within one week, the small erythema spread laminarly, exhibiting a red edge with a paler, faded centre, and the patient showed symptoms resembling an EM caused by *B. burgdorferi *(Figure 1) as an early symptom of Lyme disease [1,4]. In distinction to a typical EM with its regular, oval form, the shape of the erythema was nearly rectangular and displayed "protuberances" at the edge of the lesion (Figure 1). Thus, the clinical picture, together with the serological findings did not clearly correlate with the early clinical manifestation of Lyme disease, namely EM [2,3]. Since the patient complained about a persistent pruritus, which is atypical in EM (where minimal pruritus is only occasionally described at an early stage), a dermal mycosis was suspected, leading to the prescription of a topical antimycotic drug solution with the active component Econazol (Janssen Cilag GmbH, Neuss, Germany) by the family physician. The treatment proved to be ineffective since the erythema showed clinical progression and started to cover the complete right chest, neck and shoulder. It still presented a clear erythematous edge and a faded centre (Figure 1).

**Figure 2:**
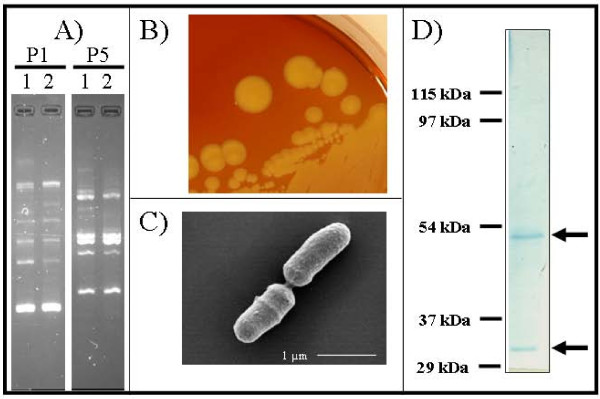
**Analysis of *Arthrobacter mysorens***. A) Randomly amplified polymorphic DNA (RAPD) analysis of the clinical isolate (lane 1) and the soil isolate (lane 2) using primer P1 [5'-GGTGCGGGAA-3'] and primer P5 [5'-AACGCGCAAC-3']. The analyses were done with the "Ready To Go RAPD analysis beads" test kit from Pharmacia (Fribourg, Germany). B) Yellow-pigmented *A. mysorens *clinical isolates grown on sheep blood agar plates after an incubation period of 72 hours at room temperature. C) Scanning electron microscopy of A.mys*orens *clinical isolates. Magnification: 30,000 ×. D) Immunoblot analysis of the patient's serum using a whole cell extract of *A. mysorens *clinical isolate. Arrow heads indicate the two reacting proteins of ~53-kDa and ~32-kDa, respectively.

At this point, the family consulted the Institute of Medical Microbiology (Giessen, Germany) and as part of a staged diagnostic approach, three swabs of the skin were taken simultaneously from different localisations; the first one from the initial focus of infection, the second one from the edge of the erythema and the third one approximately 50 millimetres outside the visible edge. The swabs were cultured for fungi and bacteria. The culture on sheep blood agar plates from swab one and two revealed many yellow-pigmented small colonies at room temperature after 48 hours of incubation. After 72 to 96 hours, the colonies showed a frayed edge indicating motile bacteria (Figure 2). These colonies could not be demonstrated in the culture of the third swab from the outside of the erythema. Additionally, few *Staphyloccus epidermidis *colonies, as members of the indigenous skin flora, could be found in all the three swab samples. No fungal growth could be evidenced even after prolonged incubation of 4 weeks, which argues against the presence of dermatophytes. The yellow-pigmented colonies did not grow at 37°C in the presence or absence of CO_2_. The partial sequencing of the 16 S rRNA gene and data base analysis using the BLAST algorithm (NCBI) and the SepsiTest™ BLAST http://www.sepsitest-blast.de identified the isolate as *Arthrobacter mysorens *(GeneBank accession number HM751094). Biochemical assays using API systems (BioMerieux, Nuertingen, Germany) demonstrated only the genus *Arthrobacter *but not the species. Antibiotic susceptibility testing via agar diffusion method and E-Test, classified the isolate to be susceptible against amoxicillin, doxycycline, cefuroxime, ceftriaxone, and cefotaxime (MIC's < 0.064 mg/ml). The patient was thereafter successfully treated with oral amoxicilline for one week (3 × 1 g per day). The skin erythema showed significant clinical improvement and was no longer detectable within two days. Subsequent swab analysis remained negative for *Arthrobacter mysorens*. To further determine the role of *A. mysorens *an immunoblot analysis using a whole cell extract of *A. mysorens *was performed. The patient's serum showed a specific immune response against a ~53-kDa and a ~32-kDa protein, respectively (Figure 2D), which demonstrates that the immune system responded to *A. mysorens *with a type-specific response. A control *Borrelia *serology after 6 months demonstrated a loss of the OspC and p18 bands and a weakened intensity of the BmpA band in the IgG-immunoblot, thus indicating a diminishing immune response and supporting the initial theory of a residual antibody response reflecting a past infection. The genus *Arthrobacter *includes a heterogenous group of aerobic, gram-positive, catalase-positive, non-fermentative coryneform bacteria that are widely distributed in the environment where their main habitat is the soil. Cells are able to resist desiccation and starvation [7]. From a review of the literature only about 42 documented cases of *Arthrobacter *species isolated from clinical samples could be retrieved [8].

## Conclusions

To our knowledge, this is the first documented case of a skin infection with *A. mysorens*, probably reflecting a new relevant human clinical isolate of this genus. In order to identify the possible source of the infection, we collected forest soil samples (n = 50) from the area where the infection most likely occurred and could indeed isolate *A. mysorens *from one sample. Randomly amplified polymorphic DNA (RAPD) analysis using two different primers revealed clonal identity between the soil and the skin isolate (Figure 2A) [9]. Therefore, the localised skin infection caused by this particular soil bacterium was likely to be caused by contamination with forest soil. The mobile pathogen (Figure 2) seems to be capable of epidermal spread and could be detected only in the centre and at the edge of the erythema (Figure 1). *Arthrobacter *species have been occasionally isolated from patients with immunodeficiencies [7]. Furthermore, *Arthrobacter *species have been described as microbial allergens which can cause allergic reactions in furniture factory workers and in people occupationally exposed to herbal dust [10,11]. The ability of *Arthrobacter *species to cause allergic reactions is a plausible explanation for the clinical symptom of pruritus described by the patient.

Because of the difficulties in culturing and identifying *Arthrobacter *isolates by conventional culture methods and biochemical assays, it is likely that infections with these coryneform bacteria are underreported, especially as the standard treatment regime for EM (doxycyline, amoxicillin, cefuroxime axetil) would also treat *Arthrobacter *infections. Cultivation of these bacteria was possible by using incubation at room temperature, which is in general not performed or recommended for these types of samples. Therefore, we advise incubation at room temperature and prolonged incubation for the bacterial culture of skin samples derived from erythema and, in case of bacterial growth the use of molecular diagnostic techniques like 16 S rRNA gene sequencing or MALDI-TOF MS for the identification of unusual bacterial isolates. This report shows the importance of clearly distinguishing such an infection from the well-described early manifestation of Lyme disease, namely EM, after a tick bite.

## Competing interests

The authors declare that they have no competing interests.

## Authors' contributions

**CI **carried out the serological studies, did molecular diagnostics, contributed to the discussion of the results and drafted and wrote the manuscript. **TH **carried out the sequencing and the bioinformatic analysis. **HH **carried out the interpretation of the serological results and contributed to the discussion of the results. **TC **contributed to the assessment and discussion of the results. **ED **carried out the design of the case report, collected the patient samples, examined the forest area, collected the soil samples, generated the figures, contributed to the assessment and discussion of the results and drafted and wrote the manuscript. All authors read and approved the final manuscript.

## Consent

Written informed consent was obtained from the patient's parents for publication of this case report and any accompanying images. A copy of the written consent is available for review by the Editor-in-Chief of this journal.

## Pre-publication history

The pre-publication history for this paper can be accessed here:

http://www.biomedcentral.com/1471-2334/10/352/prepub

## References

[B1] Nau R Christen HJ Eiffert H Lyme disease-current state of knowledge Dtsch Arztebl Int 2009 106 72 81 19562015 10.3238/arztebl.2009.0072 PMC2695290

[B2] Dandache P Nadelman RB Erythema migrans Infect Dis Clin North Am 2008 22 235 60 10.1016/j.idc.2007.12.012 18452799

[B3] Stanek G Strle F Lyme disease: European perspective Infect Dis Clin North Am 2008 22 327 39 10.1016/j.idc.2008.01.001 18452805

[B4] Bratton RL Whiteside JW Hovan MJ Engle RL Edwards FD Diagnosis and treatment of Lyme disease Mayo Clin Proc 2008 83 566 71 10.4065/83.5.566 18452688

[B5] McGinley-Smith DE Tsao SS Dermatoses from ticks J Am Acad Dermatol 2003 49 363 92 10.1067/S0190-9622(03)01868-1 12963900

[B6] Wilske B Zöller L Brade V Eiffert H Göbel UB Stanek G Quality standards for the microbiological diagnosis of infectious diseases (number 12): Lyme borreliosis Urban & Fischer Verlag, Munich, Germany 2000

[B7] Funke G Bernard KA Murray PR, BaronEJ, Pfaller MA, Jorgensen JH, Yolken RH Coryneform gram-positive rods Manual of clinical microbiology 2003 8 ASM Press, Washington, D.C 472 501

[B8] Bernasconi E Valsangiacomo C Peduzzi R Carota A Moccetti T Funke G *Arthrobacter woluwensis *subacute infective endocarditis: case report and review of the literature Clin Infect Dis 2004 38 27 31 10.1086/381436 14765360

[B9] van Belkum A Struelens M de Visser A Verbrugh H Tibayrenc M Role of genomic typing in taxonomy, evolutionary genetics, and microbial epidemiology Clin Microbiol Rev 2001 14 547 60 10.1128/CMR.14.3.547-560.2001 11432813 PMC88989

[B10] Golec M Skorska C Mackiewicz B Dutkiewicz J Immunologic reactivity to work-related airborne allergens in people occupationally exposed to dust from herbs Ann Agric Environ Med 2004 11 121 7 15236509

[B11] Skorska C Krysinska-Traczyk E Milanowski J Cholewa G Sitkowska J Gora A Dutkiewicz J Response of furniture factory workers to work-related airborne allergens Ann Agric Environ Med 2002 9 91 7 12088404

